# Downstream Impact of a Sleep Surgery Program; Optimizing Patient Care and Generating Clinical Volume

**DOI:** 10.1002/lary.70272

**Published:** 2025-11-26

**Authors:** Sruti Tekumalla, Praneet Kaki, Joseph Lu, Maurits Boon, Colin Huntley

**Affiliations:** ^1^ Department of Otolaryngology—Head & Neck Surgery Thomas Jefferson University Philadelphia Pennsylvania USA; ^2^ Sidney Kimmel Medical College Thomas Jefferson University Philadelphia Pennsylvania USA

**Keywords:** obstructive sleep apnea, otolaryngology, sleep apnea, sleep medicine, sleep surgery

## Abstract

**Objectives:**

In recent years, a growing number of Otolaryngologists have incorporated the management of sleep disorders into their practice. We aim to assess the downstream impact of a Sleep Surgery division within a department of Otolaryngology.

**Methods:**

We assess all new patients with a diagnosis of sleep apnea presenting to our division of Sleep Surgery, consisting of two surgeons, from January 1, 2021 through December 31, 2021. Our outcome measures included testing and procedures that occurred because of our workup of new patients presenting to our program. We exclusively assessed professional reimbursement of our patients insured by Centers for Medicare and Medicaid Services (CMS) through the Physician fee schedule website and modeled professional reimbursement of those with private insurance.

**Results:**

Six hundred and seventeen new patients were seen during the study period. One hundred and sixty‐six of these were insured through CMS. In evaluation and treatment of these patients, 298 sleep studies were performed on our study cohort. Three hundred and thirty‐three patients underwent Drug Induced Sleep Endoscopy (DISE). Two hundred and twenty‐eight patients underwent a surgical procedure for the management of their OSA. Four hundred and ninety consultations with other subspecialists were completed. One hundred and sixty‐one patients elected not to pursue surgical intervention and were referred to sleep medicine where they returned to CPAP. Using the CMS physician fee website, we found $129,589.77 in professional reimbursements. Financial modeling estimated $901,504.63 in professional reimbursements for those with private insurance.

**Conclusion:**

The establishment of a robust Sleep Surgery program with the ability to offer patients alternative options to CPAP provides significant downstream benefits to both patients and an institution.

**Level of Evidence:**

N/A.

## 1 Introduction

Obstructive sleep apnea (OSA) is a disease characterized by recurrent episodes of partial or complete upper airway obstruction during sleep. The obstructive episodes lead to disturbances in sleep along with ventilatory disruption causing alterations in oxygenation, carbon dioxide levels, and activation of the sympathetic nervous system. Patients with OSA commonly complain of loud snoring, poor sleep quality, excessive daytime sleepiness, and fatigue throughout the day. Untreated sleep apnea has also been associated with an increased risk of hypertension, coronary artery disease, cerebrovascular disease, cardiac arrhythmia, among other comorbidities [1, 2, 3].

There are numerous options for the management of OSA. However, the use of positive pressure (PAP) therapy is the typical initial treatment modality. When defined as use of PAP for ≥ 4 h per night, 46%–83% of patients have been reported to be nonadherent to therapy [4]. As patients are followed longitudinally, use of CPAP diminishes and a large percentage of patients are lost to follow‐up. Gabryelska et al. reviewed 400 patients over 43 months. At the study end point, 35% of the population was regularly using CPAP with 41% either failing to initiate PAP therapy or lost to follow‐up [5].

For decades, the management of OSA has included alternative treatment options to PAP and involved surgical subspecialists. In the 1970s, tracheostomy was described for the management of sleep‐disordered breathing. This offered significant benefit for disease mitigation, but can be associated with surgical complications and meaningful social stigma [6, 7]. In 1981, Fujita introduced uvulopalatopharyngoplasty (UPPP) which offered benefit for many patients with OSA and was a more socially acceptable option compared to tracheostomy [8].

Since Fujita's original article, multiple new pharyngoplasty techniques have been described [9, 10, 11, 12, 13, 14, 15]. Methods to address tongue base obstruction have also progressed as technology and techniques have evolved and improved [9, 10, 11, 12, 13, 14, 15, 16, 17, 18, 19, 20].

Hypoglossal nerve stimulation (HGNS) has emerged as an additional option. There are two HGNS devices currently approved for use by the FDA: Inspire Medical Systems, Golden Valley, MN and Nyxoah, Mont‐Saint‐Guibert, Belgium. The stimulation therapy for apnea reduction (STAR) trial was the pivotal FDA approval study for the Inspire HGNS device which showed significant improvements in both apnea reduction and symptom control [21]. The DREAM trial was the pivotal study assessing the safety and effectiveness of the Nyxoah Genio device [22]. There is one other HGNS system currently in existence (LivaNova; Houston, TX) undergoing clinical trial and awaiting FDA evaluation.

PAP is a good option for the management of OSA, but with many patients intolerant and nonadherent, alternative treatment strategies are needed. With this study, we evaluate the downstream benefits of a Sleep Surgery program, offering alternative strategies for treatment. To our knowledge this is the first evaluation in the literature of this topic. We hypothesize that a Sleep Surgery program will attract and provide treatment for a significant number of patients seeking management of their OSA. This will likely generate numerous sleep studies, consultations to other specialties, and surgical procedures.

## 2 Methods

This study was approved by our university's Institutional Review Board (iRISID‐2023‐2168).

We performed a retrospective chart review of all patients presenting for a new patient visit (NPV) for sleep apnea from January 1, 2021 through December 31, 2021 to the Sleep Surgery clinic in the Thomas Jefferson University Department of Otolaryngology‐Head & Neck Surgery. Each surgeon (CH and MB) was working at a clinical FTE of 0.8 during the study period which represented on average 3 to 4, ½‐day clinic sessions per week with the remainder of time spent in the operating room. During the study period, each surgeon was seeing sleep patients along with patients with other primary Otolaryngologic complaints thus diluting the total number of primary sleep patients. In addition, we do not prescreen patients based on BMI, OSA disease severity, or other patient‐specific factors. This may further dilute the downstream surgical volume by including patients with mild disease, elevated BMI, or other factors precluding surgery as a favorable recommendation.

We recorded demographic and descriptive information including date of new patient visit, visit description, age at visit, gender, body mass index, home address, distance traveled to our institution, median income of their home zip code, referral status from our Sleep Medicine Department, and loss to follow‐up status from our Sleep Medicine Department. We defined loss to follow‐up from Sleep Medicine as patients who were previously seen, but with no follow‐up within 18 months prior to presentation to our office and no direct referral from Sleep Medicine.

Our outcome measures included sleep testing, additional testing, and surgical procedures which occurred because of our workup or recommended intervention after presentation to our Sleep Surgery clinic. We recorded sleep testing performed at our institution. We recorded the number of drug‐induced sleep endoscopies (DISE), nasal surgery, expansion sphincter pharyngoplasty, hypoglossal nerve stimulation, maxillomandibular advancement (MMA), and other (tonsillectomy, epiglottoplasty, and tongue base reduction) sleep procedures. We also assessed consults to other specialists and subsequent testing or procedures that resulted from presentation to our sleep surgery clinic including cardiology, oral surgery, and bariatric surgery. Patients seen during the study period were included and the testing, procedures, and other outcome measures these patients underwent were all included. Cardiology referral was largely for operative clearance prior to procedures requiring anesthesia. In addition, we recorded a history of CPAP use prior to presentation to our office. We also contacted those patients not pursuing any surgical intervention or MAD to query return to CPAP use after presentation to our office.

Given the complexity of medical billing and reimbursement, complete financial data was not available to assess the downstream revenue benefit of our Sleep Surgery Program. However, professional reimbursement is available for patients insured by Centers for Medicare and Medicaid Services (CMS) through the Physician fee schedule website [23]. We utilized this data to define the professional reimbursement for evaluation and management, operative and office‐based procedures, and diagnostic testing. Our search criteria included the year 2021, the Medicare Administrative Contractor code for the state of our institution and documented the facility price. Unfortunately, professional reimbursement was not available for patients with private insurance. However, prior work has been done to compare reimbursement from private payers to CMS. Lopez et al. completed a review in 2020 in which they stated an average reimbursement from private payers of 264% that of the CMS reimbursement for outpatient hospital services [24].

Statistics were performed using the R Project for Statistical Computing [25]. *p*‐value of < 0.05 was considered statistically significant. We analyzed descriptive statistics for the demographic and descriptive variables of our population. We also analyzed descriptive statistics for the variables we used as our outcome measures. We created a parsimonious multivariable regression model using the demographic and descriptive variables as independent variables and the outcome measures as the dependent variables. This was performed to assess relationships between the demographic and descriptive variables and our outcome measures. We used variable selection methods which removed the predictors not contributing to the model.

## 3 Results

During the study period, 617 patients met our inclusion criteria and were seen for new patient consultation. This cohort consisted of 443 men and 174 women. The mean age and BMI were 56.51 ± 14.15 years and 31.07 ± 6.03 kg/m^2^. Patients traveled an average of 25.93 ± 68.63 miles to our institution. Four hundred and ninety‐five patients had undergone a trial of CPAP prior to their visit in our department. The remaining 122 presented for workup of their sleep complaints or with a diagnosis of sleep apnea, but yet to undergo a trial of PAP therapy. One hundred and sixty (25.85%) patients were referred from our Sleep Medicine division. One hundred and forty‐eight (23.91%) patients were previously seen but lost to follow‐up by our Sleep Medicine division. We defined “lost to follow‐up” as not being seen in the past 18 months. Four hundred and fifty‐three patients had private insurance while the remaining patients had government‐funded insurance (Medicare or Medicaid). 3.7% of those with government insurance were insured by Medicaid. After the new patient visit with our department and during the study period, 298 sleep studies were performed on the study cohort (197 type 3 home sleep studies and 101 type 1 polysomnograms) (Table 1).

**TABLE 1:** Demographic data of our study cohort.

Demographic data	
Gender	443 Men, 174 Women
Age	56.51 ± 14.15 years
Body mass index	31.07 ± 6.03 kg/m^2^
Distance traveled to our institution	25.93 ± 68.63 miles
Prior CPAP trial	495 patients
No prior CPAP trial	122 patients
Private insurance	453 patients
Government insurance	164 patients

*Note*: Government insurance represents Medicare and Medicaid.

Three hundred and thirty‐three (53.97%) patients underwent drug‐induced sleep endoscopy. A total of 228 upper airway surgical procedures were performed. This consisted of 29 patients undergoing expansion sphincter pharyngoplasty, 10 undergoing maxillomandibular advancement, 61 undergoing hypoglossal nerve stimulation, 95 undergoing nasal surgery (septoplasty, turbinate reduction, nasal valve repair, and/or endoscopic sinus surgery), and 33 undergoing some other type of upper airway surgery including palatine or lingual tonsillectomy.

**FIGURE 1:**
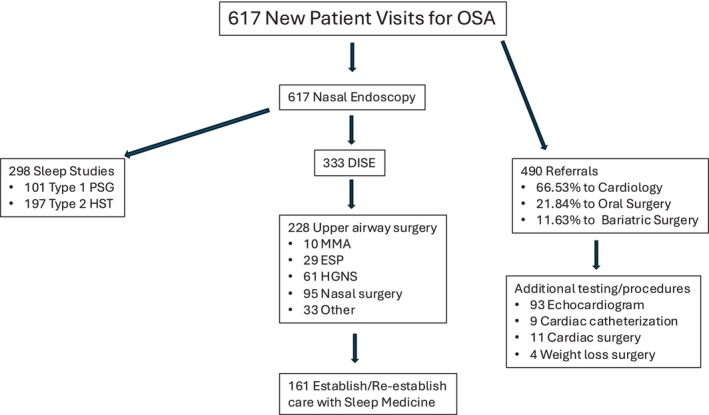
Consort style diagram depicting the flow of patients presenting for evaluation in our sleep surgery clinic. DISE, drug induced sleep endoscopy; ESP, expansion sphincter pharyngoplasty; HGNS, hypoglossal nerve stimulation; HST, Type 3 home sleep apnea test; MMA, maxillomandibular advancement; PSG, type 1 polysomnogram. [Color figure can be viewed in the online issue, which is available at www.laryngoscope.com]

Four hundred and ninety patients were referred to other specialists because of our workup. 66.53% of the referrals were to Cardiology, 21.84% to Oral maxillofacial surgery (OMFS), and 11.63% to Bariatric surgery. Of those referred to Cardiology, 93 underwent echocardiogram, nine underwent cardiac catheterization, and 11 underwent cardiac surgery. Of those referred to OMFS, 52 were referred for evaluation of MMA and 55 were referred for evaluation for a MAD. Of those referred to Bariatric surgery, four underwent gastric sleeve during the study period (Figure 1).

Patients who did not undergo surgical intervention, treatment with a MAD, or who elected to pursue weight management as a primary treatment option for OSA were contacted by phone. One hundred and sixty‐one patients had elected to reestablish care with Sleep Medicine and had returned to CPAP after their initial visit with us.

We were able to calculate the professional fee payment for the 164 patients insured by CMS. The professional fees we assessed included those for new patient visit evaluation and management, testing, office procedures, and surgical procedures. This totaled $129,589.77.

We were able to estimate the professional reimbursement for the above‐mentioned services for the 453 patients with private insurance given the above referenced private payer reimbursement rate of 264% that of the CMS (36) (Table 2). This totaled $901,504.63.

**TABLE 2:** Professional reimbursement for 164 patients insured through CMS and estimated reimbursement for 453 patients with private insurance.

Billing category	Average CMS professional fee reimbursement per charge	CMS reimbursement	Estimated private insurance reimbursement
New patient visit E&M (*n* = 617)	$143.51	$23,535.64	$171,626.48
Nasal endoscopy (*n* = 617)	$68.33	$11,206.12	$81,717.21
Type 1 polysomnography (*n* = 101)	$126.55	$4808.90	$20,279.61
Type 3 home sleep apnea testing (*n* = 197)	$47.31	$1987.02	$19,234.35
Cardiology consultation (*n* = 87)	$141.51	$6457.95	$35,615.92
Echocardiogram and cardiac catheterization (*n* = 55)	$439.57	$10,110.04	$40,953.60
Drug induced sleep endoscopy (*n* = 333)	$121.42	$11,413.48	$76,290.61
Expansion sphincter pharyngoplasty (*n* = 29)	$1433.48	$7167.40	$90,825.29
Hypoglossal nerve stimulation (*n* = 61)	$1222.73	$39,127.36	$93,611.44
Nasal surgery (*n* = 95)	$983.99	$13,775.86	$206,515.98

We also performed a sub‐analysis of patients seen in the sleep surgery clinic who were never seen by our sleep medicine division and represent a unique patient attracted by our sleep surgery program. We analyzed those either not directly referred or who were lost to follow‐up. This constituted a total of 384 patients, 105 of whom were insured by CMS. The total professional fee payment as described above for these 105 patients totaled $76,920.94. The remaining patients were insured privately and the estimated professional fee payment using the conversion factor as described above totaled $574,961.22.

Data from the parsimonious multivariable regression model using the demographic and descriptive variables as independent variables and the outcome measures as the dependent variables was also performed. These include cardiac catheterization, cardiac surgery, oral surgery consultation for MMA, oral surgery consultation for MAD, bariatric surgery consultation, and bariatric surgery. Variable selection methods were used so those variables not contributing to the model were removed.

We found that government insurance was significantly associated with patients undergoing PSG, HGNS implantation, using PAP prior to our new patient visit, and cardiology consultation. Those patients traveling from a further distance were associated with HGNS implantation. In addition, those patients whose home zip code was within 5 miles of our institution were more likely to seek cardiology consultation at our institution.

Those patients with a higher BMI were more likely to have attempted PAP prior to their NPV. In addition, those patients with a higher BMI were more likely to be referred to and seen by cardiology at our institution after their NPV with our department. Those patients with a BMI between 25 and 30 kg/m^2^ and 30 and 35 kg/m^2^ were more likely to undergo DISE and HGNS than those with a BMI less than 25 and greater than 35.

## 4 Discussion

As Otolaryngologists, we possess a unique understanding of upper airway anatomy and physiology and how that relates to obstructive events leading to OSA. We also can offer a variety of options for primary management of OSA or interventions to be used as a combination approach to optimize disease mitigation. With so many people intolerant and noncompliant with PAP therapy, it is important to offer evaluation to these patients as many of them are at risk of going untreated and being lost to follow‐up. With this study we see many patients seeking consultation in our department over the course of 1 year to assess their candidacy for treatment options other than PAP. During the study period, 333 patients underwent DISE to define their anatomy and surgical options. One hundred and thirty‐three (21.49%) underwent definitive surgical intervention to address their OSA and an additional 161 (26.01%) returned to PAP. Patients were also seen by our Bariatric and Oral surgery teams to utilize weight loss and MAD to manage their disease.

In addition, our program provides significant downstream volume for our institution. Two hundred and ninety‐eight sleep studies were done on this study population through our institution's sleep lab. Five hundred and eighty‐five procedures requiring anesthesia were performed in the operating room at our institution including DISE, surgery to primarily address the patient's OSA, nasal surgery, cardiac catheterization and cardiac surgery, and bariatric surgery. Four hundred and ninety visits occurred with consultants at our institution, including Cardiology, Oral surgery, and Bariatric medicine and surgery, because of the evaluation in our sleep surgery clinic.

To our knowledge, this represents the first study evaluating the downstream impact of a subspecialty program in Otolaryngology as our literature review did not uncover any similar studies. The evaluation of the impact of specific treatment programs on downstream volume and revenue has been evaluated and established among other specialties in medicine. Maeng et al. assessed the upstream and downstream impact of endoscopic gastrointestinal ultrasound (EUS). They defined upstream as any laboratory testing, imaging, or other institutional resource used 30 days prior to the procedure and downstream as any resource used 180 days after the procedure. They showed significant resource utilization and revenue generation as a result of this procedure [26]. Pastis et al. assessed the downstream economic impact of introducing a new endobronchial ultrasound procedure at an institution. They found that the program introduced a large proportion of new patients to the institution and the downstream revenue far exceeded the collections for the procedure alone [27].

To estimate a portion of the revenue generated through our sleep surgery program, the CMS Physician Fee Schedule was utilized to approximate the payment for professional services. We assessed the reimbursement for the E&M of our initial sleep surgery office visit, office‐based procedures in the sleep surgery clinic, surgical procedures performed by our team, sleep testing, and E&M of cardiology evaluation. This showed a total payment of $129,589.77 for these professional fees for the 164 patients insured through CMS. Using the conversion factor previously published by Lopez et al., we estimated reimbursement for these professional services for the 453 patients with private insurance of $901,504.63.(36) This is an estimation and incomplete assessment of all downstream revenue generated by our sleep surgery program as the specific reimbursement data was not available from our institution. This reimbursement data represents payments for the professional services stated, but does not provide insight into the downstream revenue generated through facility fees, operating room fees, anesthesia fees, or other hospital‐based costs. The multivariable logistic regression model shows an association with patient insurance type, patient BMI, and the distance the patient needs to travel to our institution with our outcome variables. Coverage with government insurance was significantly associated with patients undergoing PSG, HGNS implantation, using PAP prior to our new patient visit, and cardiology consultation. A possible explanation for this finding may be that most of our cohort presenting with government insurance is older and has coverage with Medicare. Since Medicare coverage allows for PSG, patients with Medicare may be more likely to undergo PSG as opposed to HST. In addition, they may select HGNS over other surgical interventions due to the morbidity associated with more traditional sleep surgery and the relatively low complication rate of HGNS [28].

Those patients with higher BMI were more likely to have attempted PAP prior to their NPV. Those with a higher BMI were more likely to be referred to and seen by cardiology at our institution after their NPV with our department. This is likely secondary to the relationship between OSA and other comorbidities associated with increasing BMI [29, 30]. Those patients with a BMI between 25 and 30 kg/m^2^ and 30 and 35 kg/m^2^ were more likely to undergo DISE and HGNS than those with a BMI less than 25 and greater than 35. The inclusion criteria for HGNS include a BMI less than 35 explaining this finding.

Lastly, our analysis revealed two interesting findings. Three and eighty‐four patients were unique to our sleep program as they were never seen by or directly referred from our sleep medicine group. In addition, 161 patients elected to return to CPAP after consultation with our sleep surgery team. During the study period, these patients were referred to sleep medicine for CPAP management. Since that time, our group has been offering CPAP to patients who prefer to follow up with our team [31]. Many of the patients returning to CPAP require an updated sleep study and our protocol is to follow up with them after the sleep study and then again at least twice over the next 12 months to assess CPAP outcomes, allowing for further downstream clinical volume. It is possible that many of the patients unique to our program would not have pursued evaluation at our institution without our program. One hypothesis for this finding is that patients can make a more informed decision about their individual treatment after pursuing a comprehensive evaluation and being presented with both medical and surgical treatment options. Both findings require further study and are topics for future research.

This study has several limitations. It was designed retrospectively, and the study period only encompasses 1 year. This limits the data available to that recorded in the medical record and the short study period does not allow for potential variations in patient flow over time. Our data also only evaluates patients presenting as new patients to the program, not established patients, which limits the overall assessment of downstream revenue. In addition, the study period was at the end of the COVID‐19 pandemic which may have impacted patient decision making on office evaluation or surgical intervention and blunted the clinical volume. Because of the retrospective design, we can collect data on patients undergoing treatment at our institution, but not those seeking recommended testing or treatment elsewhere. The senior authors were also seeing patients with non‐sleep Otolaryngologic complaints at the time of the study. A practice solely devoted to sleep would likely generate more downstream sleep‐related volume. We also do not prescreen patients prior to a new visit based on disease severity, BMI, or other patient‐specific factors. Incorporation of a screening program and addition of an APP to see those patients unlikely to be initial surgical candidates may allow the surgeon to be more surgically productive. In addition, we do not present outcome data here. This is beyond the scope of this project but provides an opportunity for future study to assess not only patient volume, but treatment effect. Lastly, we present data on downstream volume, but do not have accurate data on downstream revenue. An estimation of professional fees was assessed through publicly available sources but represents only a partial picture. Despite our efforts, we have been unable to collect this data on patients in this cohort. A prospectively designed follow‐up study may allow for a more accurate collection of charge codes and evaluation of downstream revenue.

## 5 Conclusion

The establishment of a robust Sleep Surgery program with the ability to offer alternative options to patients with OSA struggling to tolerate PAP therapy provides significant downstream benefit. It can attract many new patients into the institution and provide treatment for those struggling to manage their disease. In addition, it can generate significant downstream volume for an institution through sleep testing, surgical procedures, and subspecialty consultation.

As new options for OSA management are developed, patients become better educated about their disease and treatment options, otolaryngologists are likely to have an expanding role in the management of OSA.

## Funding

The authors have nothing to report.

## Conflicts of Interest

Colin Huntley, MD has research support from Nyxoah and Inspire Medical. He serves as a consultant for Nyxoah Medical, Inspire Medical, and Avivomed. Maurits Boon, MD serves as the Chief Medical Officer for Nyxoah.

## References

[1] K. M. Hla , T. Young , E. W. Hagen , et al., “Coronary Heart Disease Incidence in Sleep Disordered Breathing: The Wisconsin Sleep Cohort Study,” Sleep 38, no. 5 (2015): 677–684. 25515104 10.5665/sleep.4654 PMC4402672

[2] M. Arzt , T. Young , L. Finn , J. B. Skatrud , and T. D. Bradley , “Association of Sleep‐Disordered Breathing and the Occurrence of Stroke,” American Journal of Respiratory and Critical Care Medicine 172, no. 11 (2005): 1447–1451. 16141444 10.1164/rccm.200505-702OC PMC2718439

[3] T. Young , M. Palta , J. Dempsey , P. E. Peppard , F. J. Nieto , and K. M. Hla , “Burden of Sleep Apnea: Rationale, Design, and Major Findings of the Wisconsin Sleep Cohort Study,” WMJ: Official Publication of the State Medical Society of Wisconsin 108, no. 5 (2009): 246–249. 19743755 PMC2858234

[4] T. E. Weaver and R. R. Grunstein , “Adherence to Continuous Positive Airway Pressure Therapy,” Proceedings of the American Thoracic Society 5, no. 2 (2008): 173–178. 18250209 10.1513/pats.200708-119MG PMC2645251

[5] A. Gabryelska , M. Sochal , B. Wasik , P. Szczepanowski , and P. Białasiewicz , “Factors Affecting Long‐Term Compliance of CPAP Treatment—A Single Centre Experience,” Journal of Clinical Medicine 11, no. 1 (2021): 139. 35011878 10.3390/jcm11010139 PMC8745469

[6] W. J. Fee and P. Ward , “Permanent Tracheostomy: A New Surgical Technique,” Annals of Otology, Rhinology and Laryngology 86 (1977): 635–638. 334025 10.1177/000348947708600517

[7] E. D. Weitzman , C. P. Pollack , and B. Borowiecki , “Hypersomnia‐Sleep Apnea due to Micrognathia: Reversal by Tracheoplasty,” Archives of Neurology 35, no. 6 (1978): 392–395. 207245 10.1001/archneur.1978.00500300066013

[8] S. Fujita , W. Conway , F. Zorick , and T. Roth , “Surgical Correction of Anatomic Abnormalities in Obstructive Sleep Apnea Syndrome: Uvulopalatopharyngoplasty,” Otolaryngology‐Head and Neck Surgery 89, no. 6 (1981): 923–934. 6801592 10.1177/019459988108900609

[9] C. Vicini , E. Hendawy , A. Campanini , et al., “Barbed Reposition Pharyngoplasty (BRP) for OSAHS: A Feasibility, Safety, Efficacy and Teachability Pilot Study. “We Are on the Giant's Shoulders.”,” European Archives of Oto‐Rhino‐Laryngology 272, no. 10 (2015): 3065–3070. 25864183 10.1007/s00405-015-3628-3

[10] G. Iannella , J. R. Lechien , T. Perrone , et al., “Barbed Reposition Pharyngoplasty (BRP) in Obstructive Sleep Apnea Treatment: State of the Art,” American Journal of Otolaryngology 43, no. 1 (2022): 103197. 34492427 10.1016/j.amjoto.2021.103197

[11] A. Moffa , V. Rinaldi , M. Mantovani , et al., “Different Barbed Pharyngoplasty Techniques for Retropalatal Collapse in Obstructive Sleep Apnea Patients: A Systematic Review,” Sleep & Breathing 24, no. 3 (2020): 1115–1127. 32350702 10.1007/s11325-020-02088-z

[12] E. B. Pang , K. P. Pang , R. C. T. Cheong , et al., “Expansion Sphincter Pharyngoplasty in OSA: A 15 Year Review,” European Archives of Oto‐Rhino‐Laryngology 280, no. 7 (2023): 3337–3344. 36859707 10.1007/s00405-023-07901-5

[13] K. P. Pang and B. T. Woodson , “Expansion Sphincter Pharyngoplasty in the Treatment of Obstructive Sleep Apnea,” Operative Techniques in Otolaryngology‐Head and Neck Surgery 17, no. 4 (2006): 223–225.

[14] K. P. Pang and B. T. Woodson , “Expansion Sphincter Pharyngoplasty: A New Technique for the Treatment of Obstructive Sleep Apnea,” Otolaryngology‐Head and Neck Surgery 137, no. 1 (2007): 110–114. 17599576 10.1016/j.otohns.2007.03.014

[15] M. B. Cahali , “Lateral Pharyngoplasty: A New Treatment for Obstructive Sleep Apnea Hypopnea Syndrome,” Laryngoscope 113, no. 11 (2003): 1961–1968. 14603056 10.1097/00005537-200311000-00020

[16] C. s. Hwang , J. w. Kim , J. w. Kim , et al., “Comparison of Robotic and Coblation Tongue Base Resection for Obstructive Sleep Apnoea,” Clinical Otolaryngology 43, no. 1 (2018): 249–255. 28800204 10.1111/coa.12951

[17] H. Y. Li , L. A. Lee , and E. J. Kezirian , “Efficacy of Coblation Endoscopic Lingual Lightening in Multilevel Surgery for Obstructive Sleep Apnea,” JAMA Otolaryngology. Head & Neck Surgery 142, no. 5 (2016): 438–443. 26987105 10.1001/jamaoto.2015.3859

[18] S. Fujita , B. T. Woodson , J. L. Clark , and R. Wittig , “Laser Midline Glossectomy as a Treatment for Obstructive Sleep Apnea,” Laryngoscope 101, no. 8 (1991): 805–809. 1865726 10.1288/00005537-199108000-00001

[19] M. A. Babademez , F. Gul , M. Sancak , and H. Kale , “Prospective Randomized Comparison of Tongue Base Resection Techniques: Robotic vs Coblation,” Clinical Otolaryngology 44, no. 6 (2019): 989–996. 31464082 10.1111/coa.13424

[20] C. Vicini , I. Dallan , P. Canzi , et al., “Transoral Robotic Surgery of the Tongue Base in Obstructive Sleep Apnea‐Hypopnea Syndrome: Anatomic Considerations and Clinical Experience,” Head & Neck 34, no. 1 (2012): 15–22. 21400628 10.1002/hed.21691

[21] Upper‐Airway Stimulation for Obstructive Sleep Apnea | NEJM, https://www.nejm.org/doi/full/10.1056/nejmoa1308659. 10.1056/NEJMoa1308659 24401051

[22] B. T. Woodson , D. T. Kent , C. Huntley , et al., “Bilateral Hypoglossal Nerve Stimulation for Obstructive Sleep Apnea: A Nonrandomized Clinical Trial,” Journal of Clinical Sleep Medicine 21 (2025): 1883–1891. 40702817 10.5664/jcsm.11822 PMC12582211

[23] Search the Physician Fee Schedule , CMS, 2024, https://www.cms.gov/medicare/physician‐fee‐schedule/search?Y=2&T=4&HT=0&CT=1&H1=42975&C=45&M=5.

[24] E. Lopez , T. Neuman , G. Jacobson , and L. Levitt , How Much More Than Medicare Do Private Insurers Pay? A Review of the Literature (KFF, 2020), https://www.kff.org/medicare/issue‐brief/how‐much‐more‐than‐medicare‐do‐private‐insurers‐pay‐a‐review‐of‐the‐literature/.

[25] T. Zhang , R Project for Statistical Computing, https://www.r‐project.org.

[26] D. Maeng , B. Wall , D. Hassen , and D. L. Diehl , “Upstream and Downstream Revenue of Upper Gastrointestinal Endoscopic Ultrasound Determined With an Episode‐Of‐Care Approach,” Endoscopy International Open 7, no. 11 (2019): E1316–E1321. 31673600 10.1055/a-0990-9458 PMC6805194

[27] N. J. Pastis , S. Simkovich , and G. A. Silvestri , “Understanding the Economic Impact of Introducing a New Procedure: Calculating Downstream Revenue of Endobronchial Ultrasound With Transbronchial Needle Aspiration as a Model,” Chest 141, no. 2 (2012): 506–512. 22315117 10.1378/chest.11-0254

[28] E. Thaler , R. Schwab , J. Maurer , et al., “Results of the ADHERE Upper Airway Stimulation Registry and Predictors of Therapy Efficacy,” Laryngoscope 130, no. 5 (2020): 1333–1338. 31520484 10.1002/lary.28286 PMC7217178

[29] Z. Dong , X. Xu , C. Wang , S. Cartledge , R. Maddison , and S. M. Shariful Islam , “Association of Overweight and Obesity With Obstructive Sleep Apnoea: A Systematic Review and Meta‐Analysis,” Obesity Medicine 17 (2020): 100185.

[30] N. Liu , J. Birstler , M. Venkatesh , L. Hanrahan , G. Chen , and L. Funk , “Obesity and BMI Cut Points for Associated Comorbidities: Electronic Health Record Study,” Journal of Medical Internet Research 23, no. 8 (2021): e24017. 34383661 10.2196/24017 PMC8386370

[31] P. Llerena , P. Kaki , J. DeKloe , et al., “Comprehensive Management of Obstructive Sleep Apnea With CPAP in a Surgical Otolaryngology Practice,” Laryngoscope 135, no. 11 (2025): 4449–4458, 10.1002/lary.32373. 40662411 PMC12645345

